# Evidence for Hydroxocobalamin in Cyanide Toxicity Caused by Smoke Inhalation: An Updated Systematic Review

**DOI:** 10.1155/emmi/1779752

**Published:** 2025-12-31

**Authors:** Wen-Yang Jin, Dao-Chao Huang, Jun Guo, Dian Jin, Ai-Fang Ying

**Affiliations:** ^1^ Emergency Department, Taizhou Hospital of Zhejiang Province Affiliated to Wenzhou Medical University, Linhai, Zhejiang, China, wmu.edu.cn; ^2^ Department of Clinical Medicine, Division 2, China Medical University, Shenyang, Liaoning, China, cmu.edu.tw

**Keywords:** cyanide poisoning, hydroxocobalamin, mortality, smoke inhalation injury, systematic review

## Abstract

**Background:**

Hydroxocobalamin is the first‐line treatment for confirmed cyanide poisoning. Its empiric use in patients with smoke inhalation injury—where cyanide toxicity is often suspected but not confirmed—remains controversial. Further research is needed to fully understand the benefits and risks associated with its use. This study was conducted in accordance with the Preferred Reporting Items for Systematic Reviews and Meta‐Analyses (PRISMA) guidelines to provide a systematic review of the use of hydroxocobalamin for the treatment of cyanide poisoning secondary to smoke inhalation injury, with a particular focus on mortality and adverse reactions.

**Methods:**

A systematic search of the Cochrane Library, PubMed, and Embase was conducted for studies on cyanide poisoning from smoke inhalation injury treated with hydroxocobalamin. The search was limited to studies from the inception of the journals until July 30, 2025. The quality of the studies was assessed using the Newcastle–Ottawa Scale.

**Results:**

Six studies, comprising a total of 1238 patients, were identified as meeting the inclusion criteria; however, they did not meet the quality threshold for meta‐analysis. Thus, only a systematic review was performed. Two studies reported mortality rates, which were found to be similar between the hydroxocobalamin and supportive treatment groups. In contrast, two studies indicated an association between hydroxocobalamin and acute kidney injury, whereas one study proposed a potential correlation with methemoglobinemia.

**Conclusions:**

In light of the uncertain benefits and potential risks associated with hydroxocobalamin use for cyanide poisoning from smoke inhalation injury, its administration should be approached with caution. Well‐designed randomized controlled trials are urgently needed to establish optimal treatment strategies for this patient population.

## 1. Introduction

Smoke inhalation injury is a major cause of morbidity and mortality among burn patients, contributing to substantial pulmonary and systemic complications [1–6]. Even without direct thermal injury, smoke exposure can trigger a cascade of pathophysiologic changes, including increased microvascular permeability, protein leakage, and pulmonary edema [3, 7]. Multiple mechanisms underlie this injury: Inhaled toxicants directly damage the respiratory epithelium [8], whereas combustion byproducts cause chemical injury to the tracheobronchial tree and alveoli [9–11]. In addition, inflammatory cascades characterized by cytokine release and neutrophil infiltration further aggravate tissue damage [12, 13]. Excess reactive oxygen species (ROS) and reduced antioxidant capacity amplify oxidative stress, ultimately leading to acute lung injury, acute respiratory distress syndrome, and pneumonia [13–18]. Despite advances in critical care, treatment remains largely supportive, and optimized management strategies are still lacking [3].

Among the various toxicants generated during combustion, hydrogen cyanide (HCN) is particularly lethal and warrants special attention in clinical management. Hydroxocobalamin, a cobalt‐containing compound, detoxifies cyanide by binding it to form cyanocobalamin, which is excreted in urine. Although hydroxocobalamin is an approved antidote for confirmed cyanide poisoning, its efficacy in smoke inhalation–associated cyanide exposure remains uncertain. Because direct measurement of blood cyanide levels is rarely feasible in emergency or prehospital settings, hydroxocobalamin is often administered empirically in suspected cases due to its relatively favorable safety profile [6, 19–22]. European guidelines endorse its use in such scenarios [23], yet implementation within U.S. prehospital systems remains inconsistent, as reported in national EMS surveys [24].

Several observational studies have evaluated hydroxocobalamin use in patients with smoke inhalation injury; however, their findings are inconclusive, and no consensus exists regarding its clinical benefit [6, 19–24]. These uncertainties highlight ongoing challenges in diagnosing and managing smoke inhalation–associated cyanide poisoning, particularly concerning diagnostic accuracy, timing of treatment, and safety considerations.

Therefore, this systematic review aims to evaluate current evidence on hydroxocobalamin use in both prehospital and in‐hospital settings, clarify its therapeutic value, and identify key knowledge gaps to guide future research.

## 2. Materials and Methods

Two reviewers (A.F.Y. and D.J.) independently screened titles and abstracts after deduplication using EndNote 20, which was employed to import, organize, and deduplicate references. Following the initial screening, potentially eligible articles were retrieved for full‐text assessment. Reasons for exclusion were documented at each stage. Any discrepancies were resolved through discussion or adjudication by a third reviewer.

Studies were included if they (1) involved patients with documented exposure to smoke inhalation, particularly from enclosed‐space fires; (2) specifically addressed smoke inhalation injury or associated acute lung injury; and (3) evaluated the therapeutic use of hydroxocobalamin. Studies were excluded if they were nonoriginal research (e.g., animal or in vitro studies, reviews, and expert opinions); lacked a clear distinction between smoke inhalation and other injuries; or involved patients under 18 years of age. Pediatric patients were excluded due to known differences in pharmacokinetics, pathophysiological responses to toxins, and treatment outcomes, which may limit comparability with adult populations. Additional exclusion criteria included prophylactic use, dosing outside standard therapeutic ranges, coadministration of multiple antidotes, absence of key clinical outcomes (e.g., mortality and acute kidney injury [AKI]), and content mismatch upon full‐text review. To minimize duplication bias, studies with incomplete data, inconsistent reporting, or repeated patient inclusion were also excluded.

Data extraction was conducted independently by the same two reviewers using a standardized, prepiloted Microsoft Excel spreadsheet specifically designed for this review. The data extraction form included fields for study identifiers (author, year, and country), study design, population characteristics (age, sample size, and exposure), details of hydroxocobalamin administration (dose, timing, and route), and clinical outcomes, including mortality, AKI, methemoglobinemia, and other adverse events. Extracted data were cross‐verified by the reviewers for accuracy and completeness. Disagreements were resolved through consensus or by consulting a third reviewer.

Study quality was assessed using the Newcastle–Ottawa Scale (NOS) [20], which evaluates three domains: cohort selection (maximum 4 points), comparability (maximum 2 points), and outcome assessment (maximum 3 points), for a total score of up to 9 points. As summarized in Supporting Appendix B, two studies (Dépret et al. and Nguyen et al.) scored 6 points, whereas the remaining four scored 4 points, indicating generally low methodological quality.

Due to the overall low methodological quality and significant heterogeneity in study designs, populations, and outcome measures, a meta‐analysis was not conducted. Risk of bias was qualitatively assessed, focusing on selection, information, and confounding domains. To enhance the NOS‐based evaluation, we conceptually referenced the Risk of Bias in Non‐Randomized Studies of Interventions (ROBINS‐I) framework to support domain‐level bias interpretation; the ROBINS‐I tool evaluates seven domains of potential bias: (1) bias due to confounding, (2) bias in selection of participants, (3) bias in classification of interventions, (4) bias due to deviations from intended interventions, (5) bias due to missing data, (6) bias in measurement of outcomes, and (7) bias in selection of the reported result. Each domain is judged as having a low, moderate, serious, or critical risk of bias.

Although a full ROBINS‐I assessment was not feasible because of limited methodological reporting in the included studies, referencing its framework helped guide the qualitative appraisal of internal validity and strengthened the interpretation of potential biases within each domain.

Individual NOS scores and detailed appraisal criteria for each included study are presented in Supporting Appendix B. A detailed search strategy was developed using both controlled vocabulary and free‐text terms. For PubMed, the Boolean logic applied was (“smoke inhalation injuries” OR “smoke inhalation injury”) AND (“hydroxocobalamin” OR “hydroxocobalami”) AND (“cyanide” OR “hydrocyanic acid” OR “hydrogen cyanide”) AND “fire.” Similar strategies were adapted for Embase and the Cochrane Library. The complete search strings for all databases are provided in Supporting Appendix A.

This systematic review was initially considered for registration in the PROSPERO database. However, due to the substantial heterogeneity of the included studies and the lack of feasibility for a quantitative meta‐analysis, the protocol did not meet the eligibility criteria for registration. Therefore, the review was not registered.

## 3. Results

### 3.1. Study Selection and Quality Assessment

**Figure 1:**
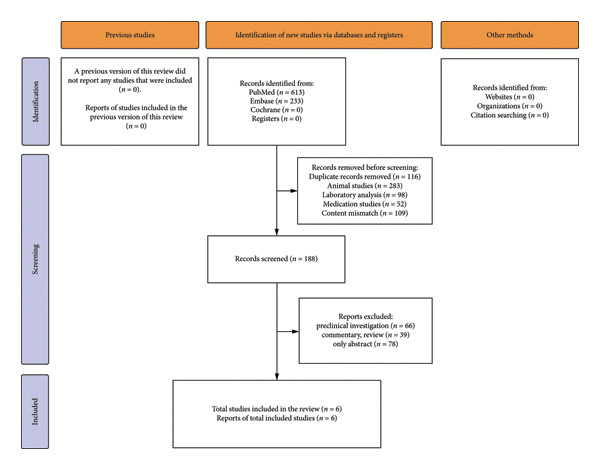
PRISMA flow diagram of study selection.

A total of 846 records were initially identified through database searches. After deduplication and screening based on the predefined inclusion and exclusion criteria, 840 records met the eligibility criteria and were included in the final analysis. The study selection process is illustrated in Figure 1. Study characteristics are summarized in Table 1. All six studies were conducted in the United States or France between 1987 and 2019, with sample sizes ranging from 21 to 739 patients. All studies involved patients with smoke inhalation injury, and several compared hydroxocobalamin treatment with standard care or no antidote. Study quality, assessed using the NOS, is summarized in Table 1 and detailed in Supporting Appendix B. Two studies (Dépret et al. and Nguyen et al.) [25, 26] scored 6, indicating moderate methodological quality, whereas the remaining four scored 4, reflecting limited comparability and selection rigor.

**Table 1:** Characteristics of the included studies on hydroxocobalamin use in smoke inhalation injury.

Study	Year	Country	Study period	Study design	Sample size	Group assignment	Mortality (%)	Adverse events
Kiernan et al.	2022	USA	2011.01.01–2019.04.30	Retrospective chart review	21	NA	67.0	Methemoglobinemia

Pruskowski et al.	2020	USA	2016.07.01–2019.04.30	Retrospective chart review	35	NA	28.9	Acute kidney injury

Dépret et al.	2019	France	2011.01–2017.12	Multicenter retrospective study	739	Hydroxocobalamin treatment group	38.1	Acute kidney injury
						No hydroxocobalamin group	27.2	

Nguyen et al.	2017	USA	2002–2008, 2008–2014	Retrospective review	273	Hydroxocobalamin treatment group	29.0	NA
						Control group without hydroxocobalamin	28.1	

Borron et al.	2007	France	1987–1994	Prospective study	69	NA	27.5	Chromaturia, pink or red skin discoloration, hypertension, erythema, and increased blood pressure

Fortin et al.	2006	France	1995–2003	Retrospective study	101	NA	41.6	Red or pink coloration of urine or skin and cutaneous rash

Abbreviations: ICU, intensive care unit; NA, not applicable; USA, United States of America.

### 3.2. Clinical Outcomes by Endpoint

#### 3.2.1. Mortality

Four studies reported mortality outcomes. In the two largest retrospective comparative studies, mortality was similar between patients who received hydroxocobalamin and those treated with standard care (Dépret et al.: 38.1% vs. 27.2%; Nguyen et al.: 29.0% vs. 28.1%) [25, 26], with no statistically significant differences observed. Other smaller observational studies reported crude mortality rates ranging from 27.5% to 67%; however, these lacked control groups and statistical adjustments, limiting interpretability.

#### 3.2.2. AKI

Two studies (Dépret et al. and Pruskowski et al.) reported a higher incidence of AKI among patients treated with hydroxocobalamin [26, 27]. Both were retrospective and lacked baseline adjustment for illness severity, leaving open the possibility of confounding and selection bias. Although these findings have raised concerns about potential nephrotoxicity, current evidence does not establish causality. The observed association likely reflects underlying hemodynamic instability or concomitant nephrotoxic exposures common in critically ill burn patients, rather than a direct toxic effect of hydroxocobalamin itself. Therefore, these results should be interpreted as hypothesis‐generating rather than confirmatory.

#### 3.2.3. Methemoglobinemia and Other Adverse Events

Methemoglobinemia was reported in one retrospective cohort (Kiernan et al.), in which 22.2% of patients exhibited elevated MetHb levels after hydroxocobalamin administration [28]. Additional self‐limiting adverse events—such as chromaturia, transient skin discoloration, and rash—were described in several studies (e.g., Borron et al. and Fortin et al.).

These outcome variations, summarized in Table 2, highlight the inconsistency of reported findings and the methodological limitations of existing studies. Well‐designed prospective studies are required to clarify both the efficacy and safety of hydroxocobalamin in patients with smoke inhalation injury.

**Table 2:** Summary of mortality, renal, and adverse outcomes reported in the included studies.

Study	Design	*n*	Mortality	AKI	MetHb	Noted bias
Dépret et al.	Retrospective	739	38.1% (vs 27.2%, NS)	↑ AKI	Not reported	Confounding, selection bias
Nguyen et al.	Retrospective	273	29.0% (vs 28.1%, NS)	Not reported	Not reported	Baseline imbalance possible
Pruskowski et al.	Retrospective	85	Not reported	↑ AKI	Not reported	Retrospective, small sample
Kiernan et al.	Retrospective	27	Not reported	Not reported	↑ 22.2% MetHb	Small cohort
Borron et al.	Observational	69	67% survival	Minor renal effects	Skin changes	No control group
Fortin et al.	Prospective	21	Not reported	Not reported	Rash, chromaturia	Limited sample, uncontrolled

## 4. Discussion

### 4.1. The Hydroxocobalamin Administration Dilemma

Hydroxocobalamin has been approved by the U.S. Food and Drug Administration (FDA) since 2006 as the first‐line antidote for confirmed cyanide poisoning. However, its empirical use in patients with smoke inhalation injury—in whom cyanide exposure is suspected but not confirmed—remains controversial due to the absence of rapid diagnostic tools and a lack of robust clinical evidence. Clinicians face significant challenges in selecting appropriate candidates, determining the optimal timing of administration—particularly in prehospital settings—and weighing the potential risks of adverse effects.

Compared with sodium nitrite, hydroxocobalamin offers a more rapid onset of action and a more favorable safety profile, particularly in patients with potential carbon monoxide coexposure, as sodium nitrite may exacerbate hypoxia [23]. For this reason, European expert consensus guidelines recommend hydroxocobalamin for suspected cyanide poisoning in victims of smoke inhalation injury [23]. However, this recommendation is primarily based on limited observational data and lacks validation from randomized controlled trials.

Further complicating clinical decision‐making, hydroxocobalamin can interfere with laboratory assays—including pulse oximetry and serum creatinine measurements—thereby hindering accurate real‐time monitoring and renal function assessment [29]. As such, its use in this setting requires cautious clinical judgment and individualized risk–benefit evaluation.

### 4.2. Hydroxocobalamin Safety

#### 4.2.1. Association With AKI

Two retrospective studies have raised concerns about a possible association between hydroxocobalamin administration and AKI. In a large multicenter cohort, Dépret et al. [26] observed a significantly higher incidence of severe AKI within 1 week following hydroxocobalamin administration, accompanied by increased vasopressor use and higher in‐hospital mortality. However, patients receiving hydroxocobalamin were generally more critically ill at baseline, suggesting that the observed relationship may partly reflect the underlying severity of illness rather than a direct nephrotoxic effect of the antidote. Several mechanisms have been proposed, including oxidative stress, pigment nephropathy, and transient alterations in renal perfusion.

Importantly, AKI is a common and multifactorial complication in critically ill populations—including those with burns, shock, or major trauma—regardless of antidote therapy. In a large ICU cohort of children and young adults [30], AKI developed in approximately 26.9% of patients. These well‐established contributors to renal injury likely coexist in smoke inhalation victims, complicating the attribution of causality to hydroxocobalamin alone.

Given this context, the reported AKI signal should be interpreted with caution. Although hydroxocobalamin may transiently affect renal biomarkers (e.g., causing falsely elevated serum creatinine due to assay interference), evidence of true structural nephrotoxicity remains inconclusive. Future studies should incorporate standardized AKI definitions (e.g., KDIGO criteria), adjust for illness severity, and distinguish between biochemical assay interference and genuine renal dysfunction. Such risk stratification is essential to determine whether hydroxocobalamin independently increases AKI risk or merely reflects the higher baseline vulnerability of critically ill patients.

Moreover, the optimal setting for empiric hydroxocobalamin administration—whether in prehospital or in‐hospital environments—remains uncertain and warrants further investigation, despite its well‐established role in confirmed cyanide toxicity.

#### 4.2.2. Association With Methemoglobinemia

Hydroxocobalamin, a vitamin B12 analog, is approved for the treatment of cyanide poisoning and is occasionally used off‐label for vasoplegic syndrome. It is not typically prescribed for B12 deficiency, for which cyanocobalamin remains the preferred agent. However, recent case reports by Kiernan et al. [28] and Jiwani et al. [31] have raised concerns about a potential link between hydroxocobalamin and administration and the development of methemoglobinemia.

Hydroxocobalamin can oxidize hemoglobin iron from ferrous to ferric forms, impairing oxygen transport and leading to methemoglobinemia—a rare but potentially serious adverse effect. Mild cases may present with cyanosis and dyspnea, whereas severe cases can result in tissue hypoxia, organ dysfunction, and potentially life‐threatening outcomes. Prompt recognition and treatment with methylene blue may be required in symptomatic patients.

Although methemoglobinemia is considered rare, its true incidence remains unclear due to limited reporting. Kiernan et al. found that 6 of 27 patients (22.2%) developed elevated methemoglobin levels after hydroxocobalamin use, peaking at a median of 7.1% around 11.4 h postadministration [28, 32]. Most cases occurred in patients with smoke inhalation exposure, with higher risk noted in those with concurrent hypoxemia or elevated carboxyhemoglobin. Given the clinical overlap between cyanide poisoning and methemoglobinemia, clinicians should remain vigilant, particularly in high‐risk scenarios [33]. Further studies are needed to clarify underlying mechanisms and preventive strategies.

### 4.3. Clinical Outcome Analysis

The efficacy of hydroxocobalamin in improving clinical outcomes following smoke inhalation injury remains uncertain. Several retrospective studies have investigated its use, yielding inconsistent findings. For example, Nguyen et al. [25] reported improved respiratory outcomes, including lower pneumonia incidence, reduced mechanical ventilation, and shorter ICU stays. In contrast, Dépret et al. [26] observed a higher incidence of AKI and no significant survival benefit, raising concerns regarding nephrotoxicity and treatment selection bias. These differences likely reflect variability in study design, baseline illness severity, and clinical protocols.

Other observational studies have described improved outcomes. Borron et al. [34] reported a 67% survival rate among 69 patients with confirmed cyanide poisoning treated with hydroxocobalamin. Hall et al. [35] highlighted its favorable safety profile and practical advantages over sodium thiosulfate in prehospital settings. However, both studies were limited by small sample sizes, lack of control groups, and uncertainty regarding cyanide exposure, which constrain generalizability.

Cross‐study comparisons are complicated by heterogeneity in diagnostic definitions, treatment timing, and the frequent coexposure to carbon monoxide. Importantly, most studies did not stratify patients by confirmed cyanide levels, making it difficult to accurately evaluate the treatment effect.

These limitations underscore the need for prospective, rigorously designed clinical trials with standardized endpoints and clearly defined control groups to determine the efficacy and safety of hydroxocobalamin in managing cyanide poisoning from smoke inhalation [36].

### 4.4. Comparative Analysis of Contrasting Studies

The studies by Dépret et al. [26] and Nguyen et al. [25] offer divergent findings that likely reflect differences in methodology and patient populations. Dépret et al. conducted a large multicenter cohort study (*n* = 739) across French ICUs, providing greater statistical power, external validity, and comprehensive follow‐up. In contrast, Nguyen et al. conducted a single‐center retrospective study (*n* = 273), limited to in‐hospital outcomes and potentially underpowered for subgroup analyses.

Patients in Dépret’s study who received hydroxocobalamin showed greater baseline illness severity, including higher vasopressor use and increased need for renal replacement therapy. This suggests possible treatment selection bias, which may have influenced the higher incidence of AKI without a corresponding survival benefit. Conversely, Nguyen et al. reported improved respiratory outcomes—such as lower pneumonia rates and shorter ICU stays—but lacked postdischarge data and did not adjust for confounders.

Taken together, these studies highlight the need for well‐controlled prospective trials using standardized treatment protocols, risk stratification, and comprehensive outcome tracking. Such research is essential to clarify the clinical utility of hydroxocobalamin in patients with smoke inhalation–associated cyanide poisoning.

### 4.5. Other Potential Risks

In addition to AKI, a possible association between hydroxocobalamin use and acute mesenteric ischemia (AMI) has been reported in burn‐injured patients, though findings remain inconclusive. Engwall et al. [37] found no evidence of AMI among patients who received hydroxocobalamin, whereas Soussi et al. [38] reported an increased risk. This discrepancy may reflect differences in study design, diagnostic criteria, or patient characteristics. Although current evidence is insufficient to establish causality, further investigation is warranted, particularly in high‐risk populations. Additionally, prehospital studies may help clarify the feasibility and safety of hydroxocobalamin use during emergency response.

### 4.6. Study Design Challenges

The current body of evidence evaluating hydroxocobalamin in smoke inhalation injury is limited by substantial methodological weaknesses. Most studies are retrospective, lack control groups, and involve small sample sizes, which constrain both the validity and generalizability. As highlighted by Borron et al. [36], Shepherd and Velez [39], and Fortin et al. [40], the absence of control arms hinders attribution of outcomes specifically to hydroxocobalamin. Moreover, in vitro and animal studies cannot replicate the complex multiorgan injury seen in human smoke inhalation, as noted by Abdullahi et al. [41].

Significant heterogeneity also exists across studies in terms of inclusion criteria, treatment timing, comparator groups, and whether hydroxocobalamin was used as a prophylactic or therapeutic agent. These inconsistencies limit the feasibility of meta‐analysis and complicate cross‐study comparisons. Additionally, few studies stratify patients based on confirmed cyanide exposure, further confounding assessments of treatment efficacy.

Smoke inhalation injuries are difficult to study due to their relatively low incidence and the absence of standardized diagnostic criteria. Nevertheless, their clinical significance is substantial—smoke exposure can increase burn‐related mortality by up to 20‐fold [42]. In mass casualty events, smoke inhalation injury is frequently observed; for example, 78% of severely burned victims from the World Trade Center attack sustained inhalation injuries.

The timing of hydroxocobalamin administration may also influence clinical outcomes. Earlier intervention has been associated with reduced mortality in cyanide poisoning [23], although specific evidence in smoke inhalation remains limited. Other potential benefits—such as cardioprotective effects [43] and use in vasoplegic shock following cardiac surgery [44]—also warrant further investigation in this context.

In conclusion, the widespread methodological variability and lack of high‐quality data necessitate rigorously designed prospective trials to clarify the efficacy, safety, and optimal use of hydroxocobalamin in patients with smoke inhalation injury.

## 5. Conclusions

Despite increasing interest, the clinical application of hydroxocobalamin in smoke inhalation injury remains inadequately understood. Current data provide insufficient clarity on its benefits or potential risks, especially in the absence of robust randomized evidence. Preliminary observations tentatively suggested that hydroxocobalamin might not confer substantial benefits to patients with smoke inhalation injuries and that its use could be associated with the incidence of AKI, potentially elevating the mortality risk facing these patients.

Smoke inhalation injury places individuals at a considerable risk of morbidity and mortality. Effectively managing these patients represents a complex and challenging conundrum for medical practitioners. The vast differences in the potential pathophysiological mechanisms relevant in patients experiencing smoke inhalation injury as compared to other sources of HCN poisoning further compound this complexity. The ethical question of whether to use potentially life‐saving treatments such as hydroxocobalamin in the absence of sufficient evidence is unresolved in medical practice. The principles of beneficence, nonmaleficence, autonomy, and justice need to be carefully balanced in the context of experimental treatments and clinical trials [45–48]. Hydroxocobalamin’s potential risks, such as AKI and methemoglobinemia, complicate its use, and healthcare providers must weigh these risks against the benefits of treating cyanide poisoning. The recommendation for future research and ethical practice is to conduct structured, high‐quality randomized controlled trials to investigate the potential benefits, risks, and outcomes of hydroxocobalamin treatment for smoke inhalation injury. These trials should be designed to minimize harm, ensure that patients receive appropriate care throughout the study, and ensure rigorous ethical oversight of clinical trials, including thorough informed consent processes that explain the potential risks and benefits to participants. In conclusion, although the use of hydroxocobalamin in treating cyanide poisoning from smoke inhalation presents significant ethical challenges, it is crucial to continue research and dialog to ensure patient‐centered, evidence‐based care.

## Conflicts of Interest

The authors declare no conflicts of interest.

## Author Contributions

Wen‐Yang Jin is the first author.

Wen‐Yang Jin, Dao‐Chao Huang, and Jun Guo contributed equally to this work and should be regarded as co‐first authors.

## Funding

This research did not receive any specific grant from funding agencies in the public, commercial, or not‐for‐profit sectors.

## Supporting Information

Additional supporting information can be found online in the Supporting Information section.

## Supporting information


**Supporting Information 1** Supporting Appendix A. Detailed database search strategies for PubMed, Embase, and the Cochrane Library.

**Supporting Information 2** Supporting Appendix B. Quality assessment of included studies using the Newcastle–Ottawa Scale.

## Data Availability

Data sharing is not applicable to this article as no datasets were generated or analyzed during the current study.

## References

[1] Gupta K. , Mehrotra M. , Kumar P. , Gogia A. R. , Prasad A. , and Fisher J. A. , Smoke Inhalation Injury: Etiopathogenesis, Diagnosis, and Management, Indian Journal of Critical Care Medicine. (March 2018) 22, no. 3, 180–188, 10.4103/ijccm.IJCCM_460_17, 2-s2.0-85044318383. 29657376 PMC5879861

[2] Tabian D. , Bulgaru Iliescu D. , Iov T. , Barna B. , Toma S. I. , and Drochioiu G. , Hydrogen Cyanide and Carboxyhemoglobin Assessment in an Open Space Fire-Related Fatality, Journal of Forensic Sciences. (2021) 66, no. 3, 1171–1175, 10.1111/1556-4029.14649. 33369895 PMC8246848

[3] Mercel A. , Tsihlis N. D. , Maile R. , and Kibbe M. R. , Emerging Therapies for Smoke Inhalation Injury: a Review, Journal of Translational Medicine. (2020) 18, no. 1, 10.1186/s12967-020-02300-4. PMC7104527 32228626

[4] Kubo T. , Osuka A. , Kabata D. , Kimura M. , Tabira K. , and Ogura H. , Chest Physical Therapy Reduces Pneumonia Following Inhalation Injury, Burns. (February 2021) 47, no. 1, 198–205, 10.1016/j.burns.2020.06.034. 32711901

[5] Deutsch C. J. , Tan A. , Smailes S. , and Dziewulski P. , The Diagnosis and Management of Inhalation Injury: an Evidence Based Approach, Burns. (August 2018) 44, no. 5, 1040–1051, 10.1016/j.burns.2017.11.013, 2-s2.0-85041634740. 29398078

[6] Foncerrada G. , Culnan D. M. , Capek K. D. et al., Inhalation Injury in the Burned Patient, Annals of Plastic Surgery. (March 2018) 80, no. 3 Suppl 2, S98–S105, 10.1097/SAP.0000000000001377, 2-s2.0-85063016615. 29461292 PMC5825291

[7] Soejima K. , Schmalstieg F. , Sakurai H. , Traber L. , and Traber D. , Pathophysiological Analysis of Combined Burn and Smoke Inhalation Injuries in Sheep, American Journal of Physiology-Lung Cellular and Molecular Physiology. (2001) 280, no. 6, L1233–L1241, 10.1152/ajplung.2001.280.6.l1233. 11350803

[8] Schick S. and Glantz S. , Philip Morris Toxicological Experiments with Fresh Sidestream Smoke: More Toxic than Mainstream Smoke, Tobacco Control. (2005) 14, no. 6, 396–404, 10.1136/tc.2005.011288, 2-s2.0-28744453551. 16319363 PMC1748121

[9] Niu Z. , Ding Z. , Chan Y. et al., Clinical Characteristics and Predictors of Burn Complicated With Smoke Inhalation Injury: A Retrospective Analysis, Experimental and Therapeutic Medicine. (November 2022) 24, no. 6, 10.3892/etm.2022.11694. PMC9748657 36561970

[10] Bingxin G. , Yichun B. , Yana M. et al., Preclinical and Clinical Studies of Smoke-Inhalation-Induced Acute Lung Injury: Update on Both Pathogenesis and Innovative Therapy, Therapeutic Advances in Respiratory Disease. (2019) 13, 10.1177/1753466619847901, 2-s2.0-85065674679. PMC6515845 31068086

[11] Wohlsein P. , Peters M. , Schulze C. , and Baumgärtner W. , Thermal Injuries in Veterinary Forensic Pathology, Veterinary Pathology Online. (September 2016) 53, no. 5, 1001–1017, 10.1177/0300985816643368, 2-s2.0-84983247547. 27106739

[12] Greven F. E. , Krop E. J. , Spithoven J. J. et al., Acute Respiratory Effects in Firefighters, American Journal of Industrial Medicine. (January 2012) 55, no. 1, 54–62, 10.1002/ajim.21012, 2-s2.0-83155173277. 21959832

[13] Albright J. M. , Davis C. S. , Bird M. D. et al., The Acute Pulmonary Inflammatory Response to the Graded Severity of Smoke Inhalation Injury, Critical Care Medicine. (April 2012) 40, no. 4, 1113–1121, 10.1097/CCM.0b013e3182374a67, 2-s2.0-84858776579. 22067627 PMC3290689

[14] Koksal G. , Oxidative Stress and Its Complications in Human Health, Advances in Bioscience and Biotechnology. (August 2012) 3, no. 8, 1113–1115, 10.4236/abb.2012.38136.

[15] Rehberg S. , Maybauer M. , Enkhbaatar P. , Maybauer D. , Yamamoto Y. , and Traber D. , Pathophysiology, Management and Treatment of Smoke Inhalation Injury, Expert Review of Respiratory Medicine. (June 2009) 3, no. 3, 283–297, 10.1586/ers.09.21, 2-s2.0-70049096501. 20161170 PMC2722076

[16] Leiphrakpam P. , Weber H. , McCain A. , Matas R. , Duarte E. , and Buesing K. , A Novel Large Animal Model of Smoke Inhalation-Induced Acute Respiratory Distress Syndrome, Respiratory Research. (2021) 22, no. 1, 10.1186/s12931-021-01788-8. PMC8261975 34233680

[17] Enkhbaatar P. and Traber D. , Pathophysiology of Acute Lung Injury in Combined Burn and Smoke Inhalation Injury, Clinical Science. (August 2004) 107, no. 2, 137–143, 10.1042/cs20040135, 2-s2.0-4043161299. 15151496

[18] Tanizaki S. , Assessing Inhalation Injury in the Emergency Room, Open Access Emergency Medicine. (2015) 7, 31–38, 10.2147/oaem.s74580, 2-s2.0-84941218374. 27147888 PMC4806805

[19] Guo G. H. and Jiang Z. Y. , [Pediatric Inhalation Injury], Zhonghua Shaoshang Zazhi. (2020) 36, no. 4, 247–251, Chinese 10.3760/cma.j.cn501120-20191002-00393. 32340413

[20] Ferrés-Padró V. , Solà-Muñoz S. , and Jimenez-Fàbrega F. X. , Evaluation of Prehospital Hydroxocobalamin Use in the Setting of Smoke Inhalation, The American Journal of Emergency Medicine. (2022) 54, 297–298, 10.1016/j.ajem.2022.01.057. 35140021

[21] Kaita Y. , Tarui T. , Shoji T. , Miyauchi H. , and Yamaguchi Y. , Cyanide Poisoning is a Possible Cause of Cardiac Arrest Among Fire Victims, and Empiric Antidote Treatment may Improve Outcomes, The American Journal of Emergency Medicine. (May 2018) 36, no. 5, 851–853, 10.1016/j.ajem.2018.01.054, 2-s2.0-85040744470. 29395761

[22] Greenhalgh D. G. , Management of Burns, New England Journal of Medicine. (2019) 380, no. 24, 2349–2359, 10.1056/NEJMra1807442, 2-s2.0-85067368130. 31189038

[23] Anseeuw K. , Delvau N. , Burillo-Putze G. et al., Cyanide Poisoning by Fire Smoke Inhalation: A European Expert Consensus, European Journal of Emergency Medicine. (February 2013) 20, no. 1, 2–9, 10.1097/MEJ.0b013e328357170b, 2-s2.0-84872177068. 22828651

[24] Purvis M. V. , Rooks H. , Young Lee J. , Longerich S. , and Kahn S. A. , Prehospital Hydroxocobalamin for Inhalation Injury and Cyanide Toxicity in the United States-Analysis of a Database and Survey of EMS Providers, Ann Burns Fire Disasters. (June 2017) 30, no. 2, 126–128. 29021725 PMC5627550

[25] Nguyen L. , Afshari A. , Kahn S. A. , McGrane S. , and Summitt B. , Utility and Outcomes of Hydroxocobalamin Use in Smoke Inhalation Patients, Burns. (February 2017) 43, no. 1, 107–113, 10.1016/j.burns.2016.07.028, 2-s2.0-84996841711. 27554631

[26] Dépret F. , Hoffmann C. , Daoud L. et al., Association Between Hydroxocobalamin Administration and Acute Kidney Injury After Smoke Inhalation: a Multicenter Retrospective Study, Critical Care. (December 2019) 23, no. 1, 10.1186/s13054-019-2706-0. PMC6929494 31870461

[27] Pruskowski K. A. , Britton G. W. , and Cancio L. C. , Outcomes After the Administration of Hydroxocobalamin, International Journal of Burns and Trauma. (2020) 10, no. 5, 231–236. 33224611 PMC7675202

[28] Kiernan E. A. , Carpenter J. E. , Dunkley C. A. et al., Elevated Methemoglobin Levels in Patients Treated with Hydroxocobalamin: A Case Series and In-Vitro Analysis, Clinical Toxicology. (2022) 60, no. 9, 1012–1018, 10.1080/15563650.2022.2072315. 35549585

[29] Dyrud M. , Niu J. , and Kohler L. J. , Elevated Methemoglobin Levels in Patients Treated With High-Dose Hydroxocobalamin, Laboratory Medicine. (2023) 55, no. 1, 50–55, 10.1093/labmed/lmad037. 37226975

[30] Kaddourah A. , Basu R. , Bagshaw S. , and Goldstein S. , Epidemiology of Acute Kidney Injury in Critically Ill Children and Young Adults, New England Journal of Medicine. (2017) 376, no. 1, 11–20, 10.1056/NEJMoa1611391, 2-s2.0-85008352251. 27959707 PMC5322803

[31] Jiwani A. Z. , Bebarta V. S. , and Cancio L. C. , Acquired Methemoglobinemia After Hydroxocobalamin Administration in a Patient With Burns and Inhalation Injury, Clinical Toxicology. (2018) 56, no. 5, 370–372, 10.1080/15563650.2017.1377838, 2-s2.0-85030538561. 28969436

[32] Wong E. , Dodson N. , Wagner S. , and Thornton S. L. , Time to Burn? Characteristics of Hydroxocobalamin Administration in an Academic Medical Center, Journal of Pharmacy Practice. (2022) 37, no. 2, 369–373, 10.1177/08971900221136633. 36318086

[33] Cumpston K. L. , Rodriguez V. , Nguyen T. et al., The Authors Respond: Prehospital Hydroxocobalamin for Smoke Inhalation, The American Journal of Emergency Medicine. (2022) 58, 323–324, 10.1016/j.ajem.2022.03.014. 35361517

[34] Trzeciak S. , Dellinger R. P. , Parrillo J. E. et al., Microcirculatory Alterations in Resuscitation and Shock Investigators. Early Microcirculatory Perfusion Derangements in Patients with Severe Sepsis and Septic Shock: Relationship to Hemodynamics, Oxygen Transport, and Survival, Annals of Emergency Medicine. (2007) 49, no. 1, 88–98, 10.1016/j.annemergmed.2006.08.021, 2-s2.0-33845872563. 17095120

[35] Hall A. H. , Dart R. C. , and Bogdan G. M. , Sodium Thiosulfate or Hydroxocobalamin for the Empiric Treatment of Cyanide Poisoning?, Annals of Emergency Medicine. (2007) 49, no. 6, 806–813, 10.1016/j.annemergmed.2006.08.022, 2-s2.0-33847185565. 17098327

[36] Hall A. H. , Saiers J. , and Baud F. , Which Cyanide Antidote?, Critical Reviews in Toxicology. (2009) 39, no. 7, 541–552, 10.1080/10408440802304944, 2-s2.0-70350496223. 19650716

[37] Engwall A. J. , Blache A. , Lintner A. , Bright A. , and Kahn S. , Hydroxocobalamin Administration After Inhalation Injury is Not Associated with Mesenteric Ischemia, Ann Burns Fire Disasters. (2021) 34, no. 3, 240–244. 34744539 PMC8534304

[38] Soussi S. , Taccori M. , De Tymowski C. et al., Risk Factors for Acute Mesenteric Ischemia in Critically Ill Burns Patients-A Matched Case-Control Study, Shock. (2019) 51, no. 2, 153–160, 10.1097/SHK.0000000000001140, 2-s2.0-85060016855. 29561390

[39] Shepherd G. and Velez L. , Role of Hydroxocobalamin in Acute Cyanide Poisoning, The Annals of Pharmacotherapy. (2008) 42, no. 5, 661–669, 10.1345/aph.1k559, 2-s2.0-42949135857. 18397973

[40] Fortin J. L. , Giocanti J. P. , Ruttimann M. , and Kowalski J. J. , Prehospital Administration of Hydroxocobalamin for Smoke Inhalation-Associated Cyanide Poisoning: 8 Years of Experience in the Paris Fire Brigade, Clinical Toxicology. (2006) 44, no. Suppl 1, 37–44, 10.1080/15563650600811870, 2-s2.0-33748987893. 16990192

[41] Abdullahi A. , Amini-Nik S. , and Jeschke M. G. , Animal Models in Burn Research, Cellular and Molecular Life Sciences. (2014) 71, no. 17, 3241–3255, 10.1007/s00018-014-1612-5, 2-s2.0-84906243920. 24714880 PMC4134422

[42] Enkhbaatar P. , Pruitt B. A.Jr, Suman O. et al., Pathophysiology, Research Challenges, and Clinical Management of Smoke Inhalation Injury, Lancet. (2016) 388, no. 10052, 1437–1446, 10.1016/S0140-6736(16)31458-1, 2-s2.0-84994881892. 27707500 PMC5241273

[43] Haouzi P. , Chenuel B. , and Sonobe T. , High-Dose Hydroxocobalamin Administered After H2S Exposure Counteracts Sulfide-Poisoning-Induced Cardiac Depression in Sheep, Clinical Toxicology. (2015) 53, no. 1, 28–36, 10.3109/15563650.2014.990976, 2-s2.0-84920101597. 25546714 PMC4332828

[44] Bak M. A. , Smith J. A. , Murfin B. , and Chen Y. , High-Dose Hydroxocobalamin for Refractory Vasoplegia Post Cardiac Surgery, Cureus. (2022) 14, no. 8, 10.7759/cureus.28267. PMC9395213 36039127

[45] Bhagwat S. and Pai S. A. , Medical Ethics in Laboratory Medicine: A Review, With an Oath for Pathologists, Indian Journal of Medical Ethics. (2020) 5, no. 1, 39–44, 10.20529/ijme.2020.02. 32103817

[46] Cameron F. , Moore B. , and Gillam L. , Two’s Company, is Three a Crowd? Ethical Cognition in Decision Making and the Role of Industry Third Parties in Pediatric Diabetes Care, Pediatric Diabetes. (2018) 20, no. 1, 15–22, 10.1111/pedi.12786, 2-s2.0-85056143380. 30311720

[47] Armitage R. , Is it Ethically Permissible for GPs to Promote Non-Directed Altruistic Kidney Donation to Healthy Adults?, Journal of Medical Ethics. (2024) 2023-109728, 10.1136/jme-2023-109728. PMC13151471 38538063

[48] Weld E. D. , Bailey T. C. , and Waitt C. , Ethical Issues in Therapeutic Use and Research in Pregnant and Breastfeeding Women, British Journal of Clinical Pharmacology. (2021) 88, no. 1, 7–21, 10.1111/bcp.14914. 33990968

