# Supplementary material for: Evidence for Hydroxocobalamin in Cyanide Toxicity Caused by Smoke Inhalation: An Updated Systematic Review
Source: Emerg Med Int. 2025 Dec 31;2025:1779752. doi: 10.1155/emmi/1779752 (PMC12767669; doi:10.1155/emmi/1779752)
Supplement: Supplementary file 1 — Supporting Information 1 Supporting Appendix A. Detailed database search strategies for PubMed, Embase, and the Cochrane Library. [file EMMI-2025-1779752-s001.docx]

# Supplementary Appendix A: Search Strategy for PubMed

Strategy and key words used to search PubMed to 30 July, 2025

| # | Search Term |
| --- | --- |
| 1 | Smoke inhalation injuries |
| 2 | Smoke inhalation injury |
| 3 | 1 OR 2 |
| 4 | Hydroxocobalamin(MeSH) |
| 5 | Hydroxocobalamin (Text Word) |
| 6 | 4 OR 5 |
| 7 | Cyanide |
| 8 | Hydrocyanic acid |
| 9 | Hydrogen cyanide |
| 10 | 7 OR 8 OR 9 |
| 11 | Fire |
| 12 | 3 AND 6 AND 10 AND 11 |

Databases and Search Strategy:

The following strategy was used to search PubMed from inception to 30 July 2025:

("smoke inhalation injuries" OR "smoke inhalation injury") AND ("hydroxocobalamin") AND ("cyanide" OR "hydrocyanic acid" OR "hydrogen cyanide") AND ("fire")
Similar search strategies were applied to Embase and the Cochrane Library using database-specific syntax.
