# Supplementary material for: Evidence for Hydroxocobalamin in Cyanide Toxicity Caused by Smoke Inhalation: An Updated Systematic Review
Source: Emerg Med Int. 2025 Dec 31;2025:1779752. doi: 10.1155/emmi/1779752 (PMC12767669; doi:10.1155/emmi/1779752)
Supplement: Supplementary file 2 — Supporting Information 2 Supporting Appendix B. Quality assessment of included studies using the Newcastle–Ottawa Scale. [file EMMI-2025-1779752-s002.docx]

# Supplementary Appendix B: Risk of Bias (Quality) Assessment Using the Newcastle-Ottawa Scale

The Newcastle-Ottawa Scale (NOS) was used to assess the methodological quality of cohort studies in three domains: Selection, Comparability, and Outcome. The maximum scores for each domain were:
Selection: 4 points
Comparability: 2 points
Outcome: 3 points
Individual study scores are summarized below.
NOS scores: S = Selection of study group; C = Comparability between groups; O = Outcome measurement
Scoring based on the Newcastle–Ottawa Scale (max scores: Selection = 4, Comparability = 2, Outcome = 3).

## Newcastle-Ottawa Quality Assessment Scale of Cohort Studies

| Author (Year) | Selection (S) | Comparability (C) | Outcome (O) | Total Score |
| --- | --- | --- | --- | --- |
| Emily Anne Kiernan et al. (2022) | 2/4 | 0/2 | 2/3 | 4 |
| Pruskowski et al. (2020) | 2/4 | 0/2 | 2/3 | 4 |
| Dépret et al. (2019) | 3/4 | 1/2 | 2/3 | 6 |
| Nguyen et al. (2017) | 3/4 | 1/2 | 2/3 | 6 |
| Borron et al. (2007) | 2/4 | 0/2 | 2/3 | 4 |
| Fortin et al. (2006) | 2/4 | 0/2 | 2/3 | 4 |
